# One-Pot Multicomponent Synthesis and Cytotoxic Evaluation of Novel 7-Substituted-5-(1*H*-Indol-3-yl)Tetrazolo[1,5-a] Pyrimidine-6-Carbonitrile

**DOI:** 10.3390/molecules25020255

**Published:** 2020-01-08

**Authors:** Mohamed A. A. Radwan, Fahad M. Alminderej, Hanem M. Awad

**Affiliations:** 1 Department of Chemistry, College of Science, Qassim University, Buraydah 51452, Saudi Arabia; falminderej@gmail.com; 2 Department of Applied Organic Chemistry, National Research Center, Dokki 12622, Egypt; 3 Tanning Materials and Leather Technology Department, National Research Centre, El-Behouth St, Dokki, Cairo 12311, Egypt; hanem_awad@yahoo.com

**Keywords:** indole, tetrazole, pyrimidine, multicomponent reaction (MCRs), cytotoxicity, HCT-116, MCF-7, MDA-MB-231, A549

## Abstract

A series of novel 7-substituted-5-(1*H*-indol-3-*yl*)tetrazolo[1,5-a]pyrimidine-6-carbonitrile was synthesized via a one-pot, three-multicomponent reaction of appropriate aldehydes, 1*H*-tetrazole-5-amine and 3-cyanoacetyl indole in catalytic triethylamine. The cytotoxic activity of the new synthesized tetrazolopyrimidine-6-carbonitrile compounds was investigated against HCT-116, MCF-7, MDA-MB-231, A549 human cancer cell lines and one human healthy normal cell line (RPE-1) using the MTT cytotoxicity assay. Compounds **4h**, **4b**, **4c**, **4i** and **4a** showed potent anticancer activities against human colon cancer. Additionally, all the compounds showed potent anticancer activities on human lung cancer.

## 1. Introduction

Indole Scaffold is one of the most extensively distributed heterocycles in nature which are widely used in drug development. The indole ring is a subunit of many natural products and medicines with extensive pharmaceutical applications [1]. Various indole-constructed heterocyclic compounds gained much attention owing to their miscellaneous biological activities, including antimicrobial, antiviral and anticancer pharmacological properties [2,3,4,5]. Within such a scaffold, tetrazole [6] and pyrimidine [7] moieties, are well-explored as a key substructure motif of several medicinal molecules as they display many biological activities such as antimicrobial [8,9], antifungal [10,11], antimalarial [12,13], anticancer [14,15], antihypertensive [16] and anti-tuberculosis. [17,18,19,20,21]. The current investigation is directed to both scaffolds, that is, tetrazole and pyrimidine moieties which could have a potential synergistic effect. This event is conducted through the general synthetic framework called Multicomponent Reactions (MCRs). In these multicomponent reactions (MCRs), three components or more interact and convert into many target products by the one-pot method [22,23,24,25,26,27]. This method is advantageous relative to mainstream synthetic frameworks in easy set-up, cost-effectiveness and high yield. Recently, several MCRs have been used effectively to build nitrogen-heterocyclic compounds especially the indole substructure that possesses distinct pharmacological and biological activities [28,29,30,31,32,33,34,35,36,37,38,39]. 

**Figure 1:**
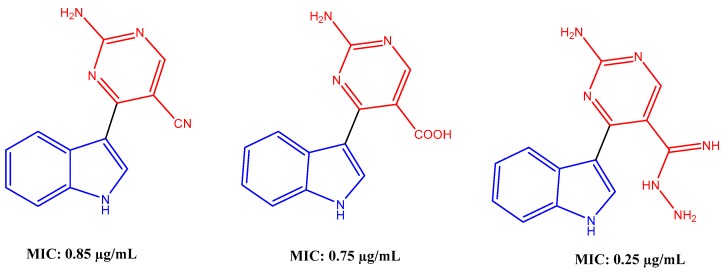
Activity of meridianin D analogs against MCF7.

**Scheme 1:**
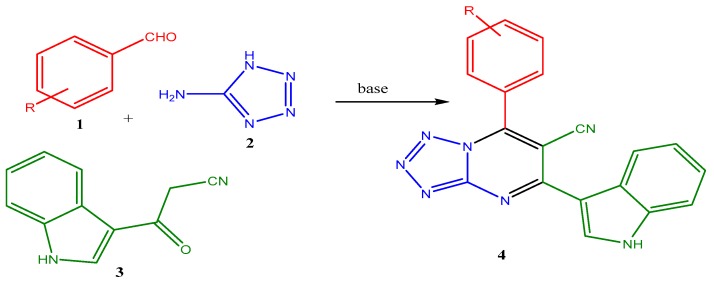
Synthesis of 7-substituted-5-(1*H*-indol-3-*yl*)tetrazolopyrimidine-6-carbonitrile.

3-Substituted indole is a nitrogen-heterocyclic compound that is predominant, and is very vital in therapeutic interactions [30,40]. Molecules with these constructions have many bioactivities, as anti-tumor [31], anti-inflammatory, reducing hypoglycemic, analgesic and antipyretic effects [30]. In continuation with our previous investigation that focused on the synthesis of bioactive indolyl-azines and azoles [41,42,43,44,45,46] (Figure 1), here we report the synthesis and cytotoxic evaluation of a series of new 7-substituted-5-(1*H*-indol-3-yl)tetrazolo[1,5-a] pyrimidine-6-carbonitrile in respectable products through MCRs reaction of 1*H*-tetrazole-5-amine, appropriate aldehydes and 3-cyanoacetyl indole (Scheme 1). 

## 2. Results and Discussion 

### 2.1. Chemistry

**Figure 2:**
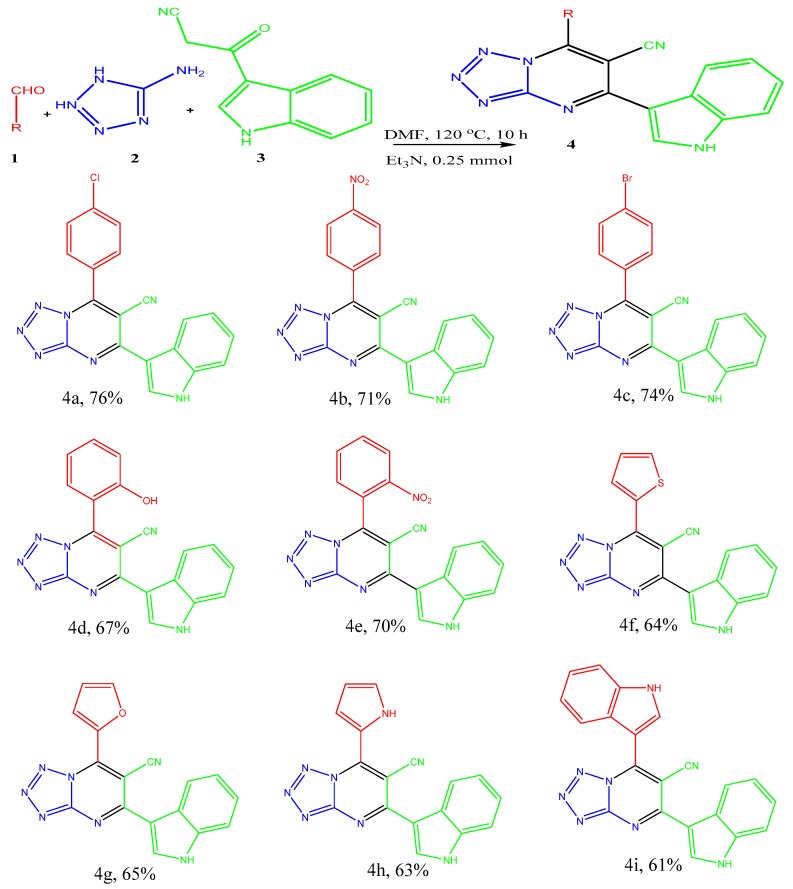
Multi-component synthesis of 7-substituted-5-(1*H*-indol-3-*yl*)tetrazolopyrimidine-6-carbonitrile. Reaction conditions: aldehyde **1** (1 mmol), 1*H*-tetrazole-5-amine **2** (1 mmol), 3-cyanoacetyl indole **3** (1 mmol), Et_3_N (0.25 mmol), in dimethylformamide (DMF) at 120 °C, and about 10 h reaction time.

In the current submission, a series of novel 7-substituted-5-(1*H*-indol-3-*yl*)tetrazolopyrimidine-6-carbonitrile was established via the multicomponent reaction of appropriate aldehydes **1**, 1*H*-tetrazole-5-amine **2** and 3-cyanoacetyl indole **3** as preliminary compounds (Scheme 1 and Figure 2). 

To improve the reaction condition, the classical MCRs were carried out in different solvents and alkali at an appropriate temperature. The MCRs were mainly performed in ethanol with no catalyst, which furnishes no products, even though it was refluxed for 48 h (entry 1, Table 1). Next, we have done the MCRs in catalytic Et_3_N in ethanol, acetonitrile, 1,4-dioxane, toluene and dimethylformamide (DMF) (entries 2–6, Table 1). Concerning the classification of the solvent, the greatest products are attained through DMF. Subsequently, we examined the MCRs in the existence of basic catalysts, for example, piperidine, 4-(dimethylamino)pyridine (DMAP), pyridine, KOH, NaOH and K_2_CO_3_ (entries 7–12, Table 1). 

Products exhibited that organic bases had a respectable catalytic result, but inorganic strong alkali (KOH, NaOH) released the less catalytic outcome. However, the inorganic weak base (K_2_CO_3_) could not supply target products. Finally, the best situation was found in this technique in DMF as a solvent, and 25 mol % triethylamine as a catalyst at 120 °C, for 10 h (Table 1). The results were recorded in Table 1. 

The construction of targets **4a–i** were recognized by the study of spectral data and mass analysis (Experimental part) as characterized for structure **4a**: the IR of **4a** unveiled absorptions at 3397 and 2223 cm^−1^ representative –NH– and cyano groups, respectively, but the amino (–NH2) and carbonyl (–C=O) signals are lost. Our ^1^H NMR chart revealed a broad singlet at δ 12.23 of –NH groups (D_2_O exchangeable) and aromatic signals at δ 7.21–8.43. The ^13^C NMR spectrum and mass spectra also supported the structural assignment (*m*/*z* 371.80, M^+^).

**Scheme 2:**
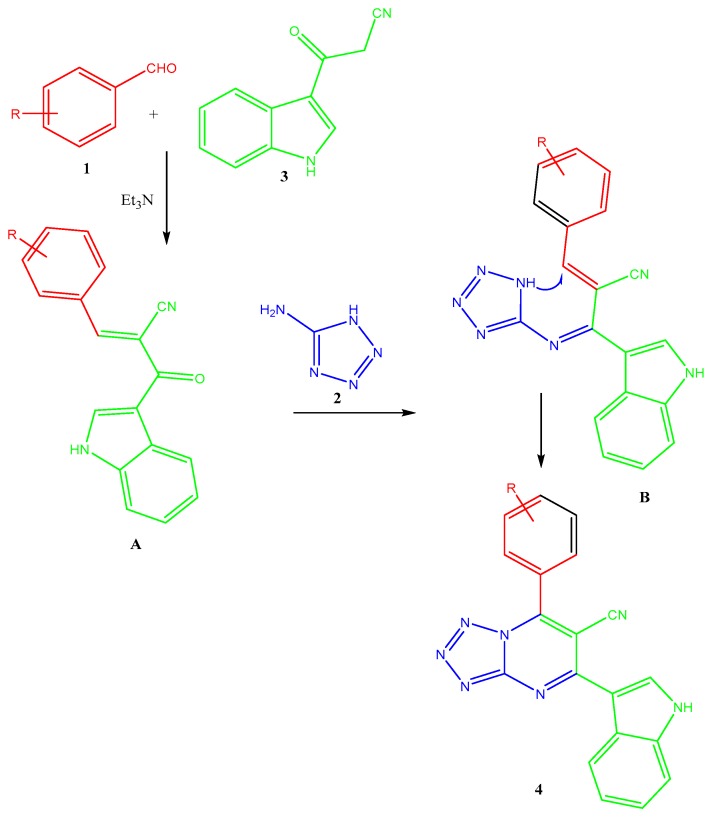
Suggested reaction mechanism.

From the above results, a probable mechanism is suggested for the production of 7-substituted-5-(1*H*-indol-3-*yl*)tetrazolopyrimidine-6-carbonitrile in the presence of triethylamine (TEA). Firstly, TEA initiates aldehydes **1** through hydrogen binding to start the nucleophilic addition of 3-cyanoacetyl indole **3**. Activation of the starting aldehydes by hydrogen bonding increases the electrophilicity of the aldehyde and supports the production of the corresponding intermediate **A** (Knoevenagel product) [47] with compound **3**. This adduct undergoes a Michael type addition reaction with 1*H*-tetrazol-5-amine **2** to yield an adduct **B** intermediate. After that, intermediate **B** underwent intramolecular cyclization leading to the C–N bond formation followed by the auto-oxidation leading to the formation product **4**, Scheme 2.

### 2.2. Cytotoxicity Screening

**Figure 3:**
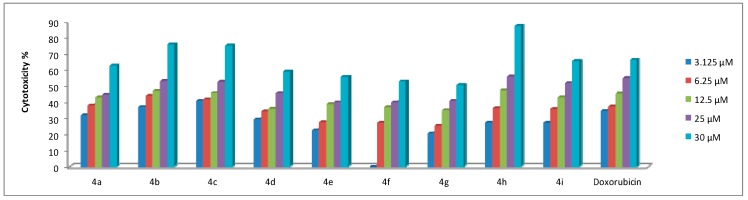
Dose dependent cytotoxic activities of new compounds on HCT-116 cancer cells according to the MTT assay.

**Figure 4:**
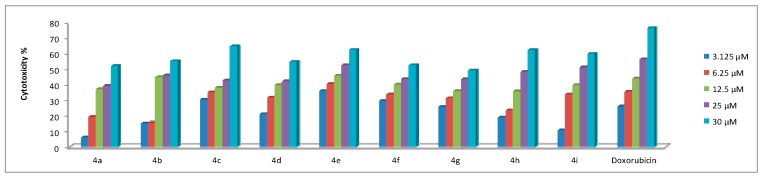
Dose dependent cytotoxic activities of new compounds on MCF-7 cancer cells according to the MTT assay.

**Figure 5:**
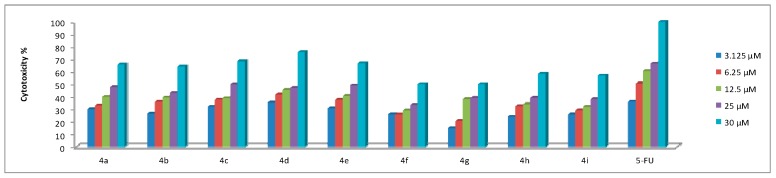
Dose dependent cytotoxic activities of new compounds on MDA-MB-231 cancer cells according to the MTT assay.

**Figure 6:**
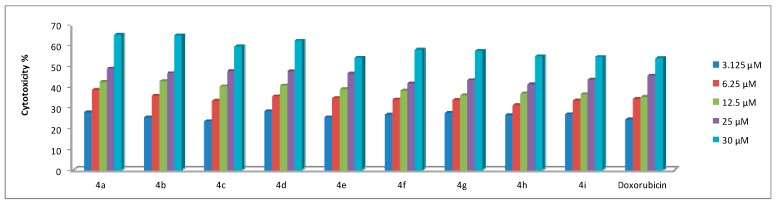
Dependent cytotoxic activities of new compounds on A549 cancer cells according to the MTT assay.

**Figure 7:**
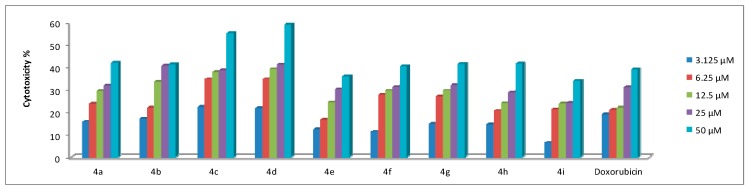
Dose dependent cytotoxic activities of new compounds on RPE-1 human normal cells according to the MTT assay.

Nine compounds **4a**–**i** were screened in vitro on HCT-116, MCF-7, MDA-MB-231 and A549 human cancer cells, as well as RPE-1 human normal cells via the MTT assay. The measurements of viable cells were considered and compared to the control. Activities of these compounds on the five human cell lines were compared to that of doxorubicin or 5-fluorouracil as reference drugs. Compounds **4a**–**i** suppressed the five human cell types in a dose-dependent manner (Figure 3, Figure 4, Figure 5, Figure 6 and Figure 7). Regarding HCT-116, both Figure 3 and Table 2 display that three compounds (**4b**, **4c** and **4h**, respectively) had more potent cytotoxic activities compared to that of doxorubicin. 

In addition, two compounds (**4i** and **4a**, respectively) had comparable cytotoxic activities and the rest of the compounds had less cytotoxic activities compared to doxorubicin. In the case of MCF-7 cells, two tested compounds (**4e** and **4c**, respectively) had slightly less anticancer activities compared to that of doxorubicin (Figure 4 and Table 2); the rest of the compounds had significantly less cytotoxic effect on MCF-7 cancer cells compared to that of the reference drug. In case of MDA-MB-231, all investigated compounds had significantly less anticancer effects compared to that of the reference drug 5-fluorouracil (Figure 5 and Table 2). In the case of A549 cancer cells, six compounds (**4a**, **4b**, **4d**, **4c**, **4e** and **4f**, respectively) had more potent cytotoxic activities; three compounds (**4g**, **4i** and **4h**, respectively) had comparable cytotoxic effect compared to that of doxorubicin (Figure 6 and Table 2). In the case of RPE-1 human normal cells, four compounds (**4d**, **4c**, **4e** and **4b**, respectively) showed more cytotoxic effect; four compounds (**4g**, **4a**, **4f** and **4h**, respectively) had comparable cytotoxic effect, and one compound (**4i**) had significantly less cytotoxic activities compared to doxorubicin. The IC_50_s indicated in Table 2 reveal that all the nine compounds have strong cytotoxic effect on cancer cells rather than on normal cells.

Furthermore, From the above-mentioned results, one can conclude that, compounds **4b**, **4c**, **4h**, **4i** and **4a** are potent anticancer candidate drugs on the human colon cancer, respectively; all the nine compounds are potent anticancer candidate drugs on the human lung cancer; compounds **4c**, **4e** and **4h** are specifically good anticancer candidate drugs on hormone-dependent human breast cancer rather than on the hormone-independent human breast cancer.

## 3. Structure–Activity Relationship

Generally, tetrazolopyrimidine unit have interesting biological activities. All synthesized compounds contain a tetrazolopyrimidine unit based on the indole ring at the 5-position. Compound **4b** substituted with 4-nitrophenyl at 7-position has the best anticancer activities against human colon cancer followed by compound **4h** which contains the pyrrole ring at the 7-position. In the case of MCF-7 cells, both compound **4e** substituted with 2-nitrophenyl at the 7-position and **4c** which contains **a** 4-bromophenyl ring at 7- had slightly less anticancer activities compared to that of doxorubicin. Compound **4a** substituted with 4-chlorophenyl at the 7-position has the best anticancer activities against A549 cancer cells. In the case of RPE-1 human normal cells, compound **4d** substituted with 2-hydroxyphenyl, followed by compound **4c** substituted with 4-bromophenyl, respectively) showed more cytotoxic effect.

## 4. Experimental

### 4.1. General Methods

All chemicals were purchased from the Merck and Sigma-Aldrich chemical companies (Muskegon, MI, USA) and used without further purification. Melting points were determined on XT-5 microscopic melting-point apparatus (Veb Analytik, Dresden, Germany) and were uncorrected. Infrared (IR) spectra were recorded on the FT Bruker Tensor 27 spectrometer ((Jasco, Easton, PA, USA). Proton nuclear magnetic resonance (^1^H NMR) and carbon-13 (C13) nuclear magnetic resonance (^13^C NMR) spectra were obtained from solution in dimethyl sulfoxide (DMSO)-*d*_6_ using a Bruker-400 spectrometer (Bruker, Karlsruhe, Germany). Mass spectra were recorded on AccuTOF LC-Plus from (Jeol, Tokyo, Japan). All melting points were determined using open capillaries on an Electrothermal-9100 (Veb, Analytik, Dresden, Germany) instrument and are uncorrected.

### 4.2. General Procedure for the Synthesis of 7-Substituted-5-(1H-indol-3-yl)tetrazolo[1,5-a]pyrimidine-6-carbonitrile ***4a**–**i***

The mixture of appropriate aldehydes (1 mmol), 1*H*-tetrazol-5-amine (1 mmol) and 3-(1*H*-indol-3-*yl*)-3-oxopropanenitrile (1 mmol) in dimethylformamide (DMF) (10 mL), Et_3_N (0.25 mmol) was put into a reaction flask and refluxed at 120 °C about 10 h (monitored by thin-layer chromatography (TLC)). After the completion of the reaction, the reaction mixture was cooled to room temperature and the products was recrystallized from DMF or ethanol (EtOH).

#### 4.2.1. 7-(4-Chlorophenyl)-5-(1*H*-indol-3-yl)tetrazolo[1,5-a]pyrimidine-6-carbonitrile **4a**

Yellow crystals; m.p.: 276–278 °C (DMF), yield (0.282 g, 76%). IR (KBr, cm^−1^): 3211 (NH), 2220 (CN), 1658, 1569, 1494, 1436, 1335, 1241, 1129, 808, 741, 684. ^1^H-NMR (400 MHz, DMSO-*d*_6_) δ (ppm): 7.21–7.46 (m, 4H, Ar-H), 7.48–7.91 (m, 4H, indole-H), 8.43 (s, 1H, indole-H_2_), 12.26 (br s, 1H, NH). ^13^C-NMR (400 MHz, DMSO-*d*_6_) δ (ppm): 150.58, 148.31, 136.80, 136.72, 136.30, 131.34, 129.23, 126.04, 123.60, 122.46, 121.30, 117.38, 113.48, 112.49, 112.09. HRMS (ESI-TOF) *m*/*z* (%) 371 (M^+^, 4), 369 (11), 270 (37), 171 (54), 146 (45), 136 (18), 117 (19), 102 (49), 86 (10), 61 (12). Anal. Calcd for C_19_H_10_ClN_7_: C, 61.38; H, 2.71; Cl, 9.54; N, 26.37. Found: C, 61.47; H, 2.66; Cl, 9.44; N, 26.23.

#### 4.2.2. 5-(1*H*-Indol-3-yl)-7-(4-nitrophenyl)tetrazolo[1,5-a]pyrimidine-6-carbonitrile **4b**

Yellow crystals; m.p.: 286–288 °C (ethanol), yield (0.273 g): 71%. IR (KBr, cm^−1^): 3232 (NH), 2223 (CN), 1655, 1561, 1550, 1463, 1430, 1332, 1243, 1127, 808, 741, 684. ^1^H-NMR (400 MHz, DMSO-*d*_6_) δ (ppm): 7.23–7.46 (m, 4H, Ar-H), 7.49–7.97 (m, 4H, indole-H), 8.48 (s, 1H, indole-H_2_), 12.33 (br s, 1H, NH). ^13^C NMR (400 MHz, DMSO-*d*_6_) δ (ppm): 149.32, 148.68, 138.64, 136.88, 136.80, 131.12,130.11, 125.96, 124.01, 122.60, 121.30, 116.77, 115.13, 113.40, 112.55. HRMS (ESI-TOF) *m*/*z* (%) 384 (M^+^ + 2, 3), 367 (5), 318 (41), 171 (64), 160 (9), 132 (8), 102 (59), 86 (100), 65 (16). Anal. Calcd for C_19_H_10_N_8_O_2_: C, 59.69; H, 2.64; N, 29.31; O, 8.37. Found: C, 59.76; H, 2.46; N, 29.21; O, 8.41.

#### 4.2.3. 7-(4-Bromophenyl)-5-(1*H*-indol-3-yl)tetrazolo[1,5-a]pyrimidine-6-carbonitrile **4c**

Yellow crystals; m.p.: 279–281 °C (ethanol), yield (0.31 g): 74%. IR (KBr, cm^−1^): 3286 (NH), 2204 (CN), 1650, 1570, 1494, 1436, 1335, 1241, 1129, 808, 741, 684. ^1^H-NMR (400 MHz, DMSO-*d*_6_) δ (ppm):7.21–7.42 (m, 4H, Ar-H), 7.44–7.90 (m, 4H, indole-H), 8.42 (s, 1H, indole-H_2_), 12.27 (br s, 1H, NH). ^13^C NMR (400 MHz, DMSO-*d*_6_) δ (ppm): 150.70, 148.14, 136.72, 136.28, 132.17, 131.98, 131.66, 126.03, 125.84, 123.60, 122.46, 121.31, 117.38, 112.50, 112.15. HRMS (ESI-TOF) *m*/*z* (%) 415 (M^+^ − 1, 4), 371 (2), 351 (19), 284 (16), 136 (18), 117 (100), 75 (31). Anal. Calcd for C_19_H_10_BrN_7_: C, 54.83; H, 2.42; Br, 19.20; N, 23.56. Found: C, 55.09; H, 2.34; Br, 19.11; N, 23.23.

#### 4.2.4. 7-(2-Hydroxyphenyl)-5-(1*H*-indol-3-yl)tetrazolo[1,5-a]pyrimidine-6-carbonitrile **4d**

Yellow crystals; m.p.: 271–273 °C (ethanol), yield (0.235 g): 67%. IR (KBr, cm^−1^): 32103324– (OH, and NH), 2210 (CN), 1620, 1560, 1492, 1433, 1334, 1241, 1129, 808, 741, 684. ^1^H-NMR (400 MHz, DMSO-*d*_6_) δ (ppm):7.22–7.41 (m, 4H, Ar-H), 7.45–7.93 (m, 4H, indole-H), 8.30 (s, 1H, indole-H_2_), 12.19 (br s, 1H, OH), 12.20 (br s, 1H, NH). ^13^C NMR (400 MHz, DMSO-*d*_6_) δ (ppm): 158.32, 154.14, 152.37, 142.20, 137.68, 136.93, 135.44, 133.12, 131.22, 126.04, 123.60, 122.46, 121.30, 117.38, 113.48, 112.49, 112.09. HRMS (ESI-TOF) *m*/*z* (%) 354 (M^+^ + 1, 2), 320 (19), 261 (15), 187 (10), 146 (45), 136 (18), 117 (19), 102 (100), 86 (10), 71 (6). Anal. Calcd for C_19_H_11_N_7_O: C, 64.59; H, 3.14; N, 27.75; O, 4.53. Found: C, 61.58; H, 2.45; Cl, 9.21; N, 26.09. 

#### 4.2.5. 5-(1*H*-Indol-3-yl)-7-(2-nitrophenyl)tetrazolo[1,5-a]pyrimidine-6-carbonitrile **4e**

Yellow crystals; m.p.: 291–293 °C (ethanol), yield (0.27 g): 70%. IR (KBr, cm^−1^): 3202 (NH), 2206 (CN), 1632, 1554, 1494, 1436, 1335, 1241, 1129, 808, 741, 684. ^1^H-NMR (400 MHz, DMSO-*d*_6_) δ (ppm): 7.20–7.39 (m, 4H, Ar-H), 7.43–7.92 (m, 4H, indole-H), 8.40 (s, 1H, indole-H_2_), 12.37 (br s, 1H, NH). ^13^C NMR (400 MHz, DMSO-*d*_6_) δ (ppm): 151.28, 147.80, 147.65, 137.64, 136.98, 136.91, 136.30, 131.34, 129.23, 126.04, 123.60, 122.46, 121.30, 117.38, 113.48, 113.12, 112.94. HRMS (ESI-TOF) *m*/*z* (%) 384 (M^+^ + 2, 20), 371 (8), 340 (49), 175 (21), 160 (11), 136 (18), 116 (100), 75(7). Anal. Calcd for C_19_H_10_N_8_O_2_: C, 59.69; H, 2.64; N, 29.31; O, 8.37. Found: C, 59.72; H, 2.39; N, 29.03; O, 8.41.

#### 4.2.6. 5-(1*H*-Indol-3-yl)-7-(thiophen-2-yl)tetrazolo[1,5-a]pyrimidine-6-carbonitrile **4f**

Yellow crystals; m.p.: 266–268 °C (ethanol), yield (0.22 g): 64%. IR (KBr, cm^−1^): 3245 (NH), 2213 (CN), 1625, 1545, 1492, 1436, 1335, 1241, 1129, 808, 741, 684. ^1^H-NMR (400 MHz, DMSO-*d*_6_) δ (ppm):7.20–7.43 (m, 3H, thiophene-H), 7.42–7.90 (m, 4H, indole-H), 8.50 (s, 1H, indole-H_2_), 12.20 (br s, 1H, NH). ^13^C NMR (400 MHz, DMSO-*d*_6_) δ (ppm): 145.42, 138.78, 136.51, 135.36, 135.20, 128.54, 126.18, 123.48, 122.30, 121.40, 118.16, 113.71, 112.43, 106.64. HRMS (ESI-TOF) *m*/*z* (%) 343 (M^+^, 3), 324 (3), 300 (20), 256 (24), 195 (18), 117 (100), 102 (70), 76 (19). Anal. Calcd for C_17_H_9_N_7_S: C, 59.47; H, 2.64; N, 28.56; S, 9.34. Found: C, 59.65; H, 2.49; N, 28.39; S, 9.21.

#### 4.2.7. 7-(Furan-2-yl)-5-(1*H*-indol-3-yl)tetrazolo[1,5-a]pyrimidine-6-carbonitrile **4g**

Yellow crystals; m.p.: 269–271 °C (ethanol), yield (0.212 g): 65%. IR (KBr, cm^−1^): 3232 (NH), 2214 (CN), 1636, 1548, 1466, 1436, 1335, 1241, 1129, 808, 741, 684. ^1^H-NMR (400 MHz, DMSO-*d*_6_) δ (ppm):7.19–7.41 (m, 3H, furan-H), 7.42–7.96 (m, 4H, indole-H), 8.44 (s, 1H, indole-H_2_),7.19–8.44 (m, 8H, Ar-H), 12.19 (br s, 1H, NH). ^13^C NMR (400 MHz, DMSO-*d*_6_) δ (ppm): 148.82, 148.76, 137.47, 135.13, 126.19, 123.48, 122.38, 122.30, 121.43, 117.75, 113.99, 113.67, 112.43, 105.70. HRMS (ESI-TOF) *m*/*z* (%) 327 (M^+^, 1), 263 (100), 223 (7), 185 (15), 160 (21), 93 (18), 86 (13). Anal. Calcd for C_17_H_9_N_7_O: C, 62.57; H, 3.09; N, 34.34. Found C, 62.69; H, 3.01; N, 34.76. 

#### 4.2.8. 5-(1*H*-Indol-3-yl)-7-(1*H*-pyrrol-2-yl)tetrazolo[1,5-a]pyrimidine-6-carbonitrile **4h**

Yellow crystals; m.p.: 258–261 °C (DMF), yield (0.21 g): 64%. IR (KBr, cm^−1^): 3231 (NH), 3221 (NH), 2216 (CN), 1658, 1569, 1494, 1436, 1335, 1241, 1129, 808, 741, 684. ^1^H-NMR (400 MHz, DMSO-*d*_6_) δ (ppm): 6.817–.41 (m, 3H, pyrrole-H), 7.42–7.90 (m, 4H, indole-H), 8.33 (s, 1H, indole-H_2_), 6.81–8.33 (m, 8H, Ar-H), 12.11 (br s, 1H, NH)11.64 (br s, 1H, NH). ^13^C NMR (400 MHz, DMSO-*d*_6_) δ (ppm): 146.98, 145.33, 136.88, 135.10, 126.12, 123.41, 122.28, 122.10, 121.12, 117.05, 113.34, 112.87, 112.21, 104.98. HRMS (ESI-TOF) *m*/*z* (%) 328 (M^+^ + 2, 1), 306 (9), 262 (11), 185 (100) (18), 159 (8), 143 (12), 82 (4). Anal. Calcd for C_17_H_10_N_8_: C, 66.45; H, 3.41; N, 30.14. Found: C, 66.59; H, 3.32; N, 30.07.

#### 4.2.9. 5,7-di(1*H*-Indol-3-yl)tetrazolo[1,5-a]pyrimidine-6-carbonitrile **4i**

Yellow crystals; m.p.: 296–298 °C (DMF), yield (0.23 g): 61%. IR (KBr, cm^−1^): 32363265– (2NH), 2206(CN), 1656, 1560, 1454, 1436, 1335, 1241, 1129, 808, 741, 684. ^1^H-NMR (400 MHz, DMSO-*d*_6_) δ (ppm): 7.17–7.94 (m, 8, di-indole-H), 8.31, 8.33 (two s, 2H, indole-H_2_), 12.01 (br s, 1H, NH), 12.23 (br s, 1H, NH). ^13^C NMR (400 MHz, DMSO-*d*_6_) δ (ppm): 151.04, 150.54 136.80, 136.72, 136.30, 131.34, 130.11, 129.23, 126.04, 123.96, 123.60, 122.46, 121.30, 117.38, 113.48, 111.22, 110.12. HRMS (ESI-TOF) *m*/*z* (%) 376 (M^+^, 4), 351 (11), 270 (37), 171 (54), 146 (45), 136 (18), 117 (19), 102 (49), 86 (10), 61 (12). Anal. Calcd for C_21_H_12_N_8_: C, 67.01; H, 3.21; N, 29.77. Found: C, 67.34; H, 3.12; N, 29.52. 

### 4.3. Materials and Methods

#### 4.3.1. In-Vitro Cytotoxic Activity

Cell cultures of human colorectal carcinoma (HCT)-116, MCF-7 (hormone-dependent human breast adenocarcinoma), MDA-MB-231 (hormone-independent human breast adenocarcinoma), A549 (human lung carcinoma) and human normal Retina pigmented epithelium (RPE)-1 cell lines were purchased from the American Type Culture Collection (Rockville, MD, USA) and maintained in Dulbecco’s modified Eagle medium (DMEM) medium which was supplemented with 10% heat-inactivated fetal bovine serum (FBS), 100 U/mL penicillin and 100 U/mL streptomycin. The cells were grown at 37 °C in a humidified atmosphere of 5% CO_2_.

#### 4.3.2. MTT Cytotoxicity Assay

The cytotoxicity activity on HCT-116, MCF-7, MDA-MB-231 and A549 human cancer cell lines as well as RPE-1 human normal cells was estimated using the 3-[4,5-dimethyl-2-thiazolyl)-2,5-diphenyl-2*H*-tetrazolium bromide (MTT) assay, which is based on the reduction of the tetrazolium salt by mitochondrial dehydrogenases in viable cells [48,49,50]. Cells were dispensed in 96-well sterile microplates (5 × 10^4^ cells/well), and incubated at 37 °C with a series of different concentrations, in DMSO, of each tested compound or Doxorubicin (positive control) for 48 h in a serum free medium prior to the MTT assay. After incubation, media were carefully removed, 40 µL of MTT (2.5 mg/mL) were added to each well and then incubated for an additional 4 h. The purple formazan dye crystals were solubilized by the addition of 200 µL of DMSO. The absorbance was measured at 570 nm using a Spectra Max Paradigm Multi-Mode microplate reader. The relative cell viability was expressed as the mean percentage of viable cells compared to the untreated control cells. The relative cell anti-proliferative was measured according to the following equation: % cytotoxicity = (1 − As/Ab) × 100. Where; As = Absorbance of each sample and Ab = Absorbance of the blank. All experiments were conducted in triplicate and repeated on three different days. All the values were represented as mean ± SD. IC_50_s were determined by probit analysis by SPSS Inc probit analysis (IBM Corp., Armonk, NY, USA).

## 5. Conclusions

Briefly, we have a conventional three-constituent reaction to the construction of a completely substituted new series of tetrazolopyrimidine-6-carbonitrile based on indole moiety in the existence of triethylamine as a catalyst and DMF as a solvent. The significance of this process is important over the additional usual ones, by short times, respectable yields, trivial conditions, cost-effective and relaxed management. Cytotoxic evaluation of novel series of 7-substituted-5-(1*H*-indol-3-yl)tetrazolopyrimidine-6-carbonitrile was investigated against HCT-116, MCF-7, MDA-MB-231 and A549 human cancer cell lines using the MTT test. From the results indicated in this work, one can conclude that compounds **4h**, **4b**, **4c**, **4i** and **4a** had potent anticancer activities against human colon cancer, respectively; all the nine compounds had potent anticancer activities on human lung cancer as well.

## Figures and Tables

**Table 1:** Optimization of the multicomponent reactions (MCRs) between 4-choloro benzaldehyde, 1*H*-tetrazole-5-amine, and 3-cyanoacetyl indole.

Entry	Base (mmol)	Solvent	Temp. (°C)	Time (h)	Yield (%)
1	-	EtOH	80	10	-
2	Et_3_N	EtOH	80	10	Trace
3	Et_3_N	CH_3_CN	80	10	12
4	Et_3_N	1,4-Dioxane	80	10	15
5	Et_3_N	Toluene	120	10	-
6	Et_3_N	DMF	120	10	76
7	Piperidine 0.5	DMF	120	10	65
8	DMAP 0.2	DMF	120	10	40
9	Pyridine	DMF	120	10	35
10	KOH	CH_3_CN	80	8	22
11	NaOH	CH_3_CN	80	8	27
12	K_2_CO_3_	CH_3_CN	80	8	Trace

**Table 2:** IC_50_ of the new compounds against the four human cancer cells as well as one human normal cell types according to the MTT assay.

Compound Code	IC50 (µM) ± SD				
	HCT-116	MCF-7	MDA-MB-231	A549	RPE-1
**4a**	20.4 ± 2.1	32.5 ± 2.4	21.1 ± 2.1	20.9 ± 2.1	61.5 ± 2.5
**4b**	14.1 ± 1.5	28.1 ± 2.1	22.5 ± 2.9	22.4 ± 1.8	56.3 ± 3.1
**4c**	14.2 ± 1.7	22.0 ± 1.8	19.4 ± 3.1	24.7 ± 2.3	42.4 ± 2.8
**4d**	23.1 ± 2.2	25.7 ± 2.1	16.2 ± 2.3	22.7 ± 2.1	31.8 ± 3.1
**4e**	26.6 ± 2.1	17.6 ± 1.5	19.7 ± 2.8	25.6 ± 1.9	50.9 ± 2.9
**4f**	31.9 ± 2.6	23.9 ± 1.9	30.6 ± 2.2	25.9 ± 2.1	62.4 ± 2.8
**4g**	28.8 ± 1.9	26.3 ± 2.5	31.1 ± 2.9	26.0 ± 2.3	60.4 ± 3.4
**4h**	14.6 ± 1.5	26.0 ± 2.1	26.3 ± 1.8	27.5 ± 2.5	67.5 ± 3.9
**4i**	19.9± 2.1	24.8 ± 2.4	27.4 ± 2.3	26.6 ± 2.1	77.2 ± 3.8
Doxorubicin	17.2 ± 1.5	17.5 ± 2.1	-	27.0 ± 2.4	66.1 ± 3.5
5-Fluorouracil	-	-	3.8 ± 0.2	-	-
